# Moderate/Severe Hyponatremia Increases the Risk of Death among Hospitalized Chinese Human Immunodeficiency Virus/Acquired Immunodeficiency Syndrome Patients

**DOI:** 10.1371/journal.pone.0111077

**Published:** 2014-10-31

**Authors:** Lijun Xu, Hanhui Ye, Fan Huang, Zongxing Yang, Biao Zhu, Yan Xu, Yunqing Qiu, Lanjuan Li

**Affiliations:** 1 The State Key Laboratory for Diagnosis and Treatment of Infectious Diseases, the First Affiliated Hospital, School of Medicine, Zhejiang University, Hangzhou City, China; 2 Collaborative Innovation Center for Diagnosis and Treatment of Infectious Diseases, the First Affiliated Hospital, School of Medicine, Zhejiang University, Hangzhou City, China; 3 Clinical Center of HIV/AIDS, Fuzhou Infectious Disease Hospital, Fujian Medical University, Fuzhou City, China; 4 Department of Infectious Diseases, Jianxin Hospital of Fujian Province, Fuzhou City, China; China Medical University, China

## Abstract

**Objectives:**

To evaluate whether the serum sodium concentration is associated with the progression and long-term prognosis of Chinese HIV/AIDS patients.

**Methods:**

Three hundred and eighty seven hospitalized patients were recruited into this retrospective cohort study. The strata of serum sodium concentration were moderate/severe hyponatremia, mild hyponatremia and normonatremia. Disease progression was estimated using CD4 counts and the WHO clinical stage. Correlation analysis was used to evaluate the serum sodium concentration with disease progression. The Kaplan-Meier method and the Cox proportional hazards model were used to analyze the effect of different serum sodium levels on survival.

**Results:**

In this study 206 patients (53.2%) had hyponatremia, including 10.6% patients with moderate/severe hyponatremia and 42.6% with mild hyponatremia. The serum sodium concentration was significantly correlated with the HIV/AIDS progression (*P*<0.001). During the follow-up, 100 patients (25.6%) died. The cumulative survival rates of HIV/AIDS patients were 47.8%±8.5% in patients with moderate/severe hyponatremia, 59.8%±5.0% with mild hyponatremia and 79.9%±3.4% with normonatremia (log-rank P<0.001). After adjusting for sex, age, WHO stage, CD4 count, hemoglobin and albumin, the relative hazard was 3.5 (95% CI: 1.9–6.5) for patients with moderate/severe hyponatremia (P<0.001), and 1.5 (95% CI: 0.9–2.4) for those with mild hyponatremia (*P* = 0.161), compared with normonatremic patients.

**Conclusions:**

The serum sodium level is closely correlated with the severity of patients. Only moderate/severe hyponatremia affects the prognosis of Chinese HIV/AIDS patients. Earlier intensive medical managements(including HAART)are necessary to increase the survival rates of Chinese HIV/AIDS patients with moderate/severe hyponatremia.

## Introduction

Acquired immunodeficiency syndrome (AIDS) is a multi-system disorder caused by defects in the immune system as a result of human immunodeficiency virus (HIV) infection that increase the vulnerability of patients to opportunistic infections, neoplasms and metabolic abnormalities [1]–[3].

Hyponatremia is one of the most common electrolyte abnormalities in AIDS patients [4]–[8], with a reported frequency of approximately 38% [5]. It has long been noted that hyponatremia is associated with pulmonary or central nervous system (CNS) infections [9]–[11], gastrointestinal sodium losses [5], adrenal insufficiency [12] and renal disorders [13]. Importantly, a CNS or pulmonary infection may result in a syndrome of inappropriate secretion of antidiuretic hormone (SIADH), which plays a pivotal role in the presentation of hyponatremia [5], [7]–[9]. Based on these observations, we hypothesized that hyponatremia is correlated with the progression of AIDS.

The determination of serum sodium concentrations is a readily available, objective, and simple reference in clinical practice. Very little is known about the association between serum sodium concentrations, CD4+ cell counts, and the severity and mortality of HIV/AIDS patients. Recent studies by Dao *et al*. indicated that a serum sodium concentration below 135 mmol/L combined with hypochloremia lead to a high first year mortality in female AIDS patients [14]. However, further investigations are required to elucidate the relationships between serum sodium concentrations and CD4+ cell counts, WHO stages and HIV-RNA levels, and whether hyponatremia is an independent risk factor for death in HIV/AIDS patients. Importantly, the effect of the serum sodium concentrations on long-term survival of AIDS patients is currently unknown.

China is experiencing an emerging HIV epidemic, primarily affecting the economically deprived populations in rural areas that are at increased risk of morbidity and mortality than the populations in urban areas [15] due the inability to afford medical care [16]. To address this issue, particularly in rural populations, the Chinese government implemented the National Free Antiretroviral Treatment Program (NFATP) in 2003, with a “**Four Free and One Care**” policy (providing free HIV blood tests; free antiretroviral treatment for AIDS patients in economic difficulties in urban and rural areas; free drugs to prevent mother-to-child transmission; free education for AIDS orphans; government-funded care for AIDS patients who live in poverty) [17]. China released the first edition of the HIV treatment guidelines in 2005. The treatment criteria were: WHO stage III/IV disease and/or CD4 cell count <200 cells/µL [17]. The treatment criteria updated in 2007 were: WHO stage III/IV disease and/or CD4 count ≤200 cells/µL, and/or CD4 count 200–350 cells/µL with HIV-RNA >100,000 copies/mL or a rapid decline in CD4 count of >100 cells/µL per year [18]. In 2012, China again modified the criteria to: WHO stage III/IV disease and/or CD4 count ≤350 cells/µL, and/or CD4 count 350–500 cells/µL with HIV-RNA >100,000 copies/mL or a rapid decline in CD4 count of >100 cells/µL per year [19]. The NFATP has made considerable progress in providing the necessary care and treatment for HIV-infected people in China. Due to demographic, geographical, and especially economic differences, some important clinical tests, such as those for HIV-RNA, are not included in this NFATP [20]; thus, the development of inexpensive and convenient clinical tests for is urgently required by those patients in poverty in China. In the present study, we investigated the correlation of sodium concentrations with the progression and prognosis of HIV/AIDS patients, aiming to identify a simple and convenient parameter to evaluate the disease severity and risk of mortality in this population.

## Materials and Methods

### Population

Between January 2006 and December 2012, 503 antiretroviral therapy (ARV)-naïve patients were hospitalized for AIDS-related complex treatment in this retrospective study. Patients were excluded if they had liver cirrhosis, thyroid disorders, congestive heart failure, renal disorders, chronic obstructive pulmonary disease (COPD), or tumor/cancer. Highly active antiretroviral therapy (HAART) was administered when the patients were in a relatively stable condition and met any of the following criteria: (i) CD4 cell count ≤200 cells//µL, (ii) WHO stage III or IV, (iii) CD4 cell count of 200–350 cells/µL with HIV-RNA >100,000 copies/mL or a rapid decline in CD4 count of >100 cells/µL per year [18], [21], [22]. The basic regimen was AZT/D4T +3TC+NVP/EFV for patients. After HAART was initiated, the patients were followed-up periodically at China's Center for Disease Control and Prevention (CDC) and as outpatients in hospital.

### Laboratory tests

Patient blood samples were drawn after a 12-h fast within 3 days of admitted to hospital. The serum sodium concentration was assayed with a biochemical autoanalyzer (Beckman Coulter, CA, USA) using an ion-selective electrode method. The numbers of CD4+ and CD8+ T cells were measured by flow cytometry (B&D Science, NJ, USA) following staining with FITC-conjugated anti-human CD4, PE-conjugated anti-human CD8, and PE-Cy5 conjugated anti-human CD3 mAbs (B&D Sciences, NJ, USA). HIV-1 RNA was assayed according to the standard protocol of the COBAS Amplicor HIV-1 Monitor Test kit (Roche, IN, USA) on the COBAS Amplicor PCR system (version 1.5) (Roche, IN, USA).

### Follow-up and clinical data collection

The initial day of follow-up was defined the day that the patient began HAART. Follow-up was performed at 6-month intervals according to a method described previously [17], [23]. Patient information was collected by hospital and local CDC staff at treatment initiation, and then at each follow-up visit, each regimen change, and again at treatment termination. The collected information was recorded using a computer that automatically converted it into an electronic database with data verification and cleaning capabilities. This real-time data collection system was able to provide information on patient demographics, laboratory test results, clinical signs and symptoms, self-reported adherence and survival data. The patients were followed-up between January 2006 and December 2012. The deadline of fellow-up was December 31, 2012; thus, the follow-up times of patients ranged from 1 week to 72 months.

### Diagnosis criteria

A serum sodium concentration ≥135 mmol/L was defined as normonatremia [11], [24]–[26]. A serum sodium concentration <135 mmol/L was defined as hyponatremia, with the level further defined as mild (serum sodium 125–134 mmol/L), moderate (115–124 mmol/L), or severe (<115 mmol/L) [27]. The progression of HIV/AIDS was evaluated on the basis of the CD4+ cell count and WHO stage (determined according to the WHO criteria) [22], [28].

### Statistical analysis

Continuous normal variables were expressed as mean ± standard deviation (SD), and categorical variables were expressed as the number of cases (percentage). CD4+ T cell counts were expressed as medians (interquartile range, IQR). HIV-RNA levels (copies/mL) were log10-transformed into variables (lg copies/mL) to meet the normality criteria for statistical analysis. Continuous variables were compared by one-way ANOVA test or Student's *t*-test, and categorical variables were compared by χ2 analysis or Fisher's exact test. Spearman's rank correlation was used to analyze the association of serum sodium concentration with the stage of HIV/AIDS. Pearson's correlation and regression analyses were used to evaluate the relationship between CD4+ T cell counts and serum sodium concentration. The effect of hyponatremia on the survival of patients was analyzed by the Kaplan–Meier method and Cox's proportional hazard model. AIDS-related death was defined as an event. Data for patients were censored at the date of last visit (for those surviving at the end of the follow-up period), or the date at which the subject was last known to be alive (for those with unknown vital status), or the date of participants in whom the cause of death was known to be unrelated to AIDS. Serum sodium concentration (mmol/L)(categories: <125, 125–134, ≥135), age (years) (categories: <30, ≥30), WHO stage (categories: I/II, III, IV), CD4 count (cells/µL) (categories: missing, 0–50, 51–200, ≥201), HIV-RNA (copies/mL) (categories: missing, <10^5^ copies/mL, ≥10^5^ copies/mL), hemoglobin (g/L) (categories: missing, <80, 80–109, ≥110) and albumin (g/L) (categories: missing, <30, ≥30) were included in the models as time-independent covariates. These covariates were first analyzed in the univarate Cox model, then the covariates with *P*-values <0.2 in the univarate model were selected for analysis in the multivariate Cox proportional hazard model using the Forward Stepwise (Likelihood Ratio) method. A *P*-value <0.05 (two-tailed) was considered to indicate statistical significance. Data analysis was performed using the SPSS 13.0 statistical software (SPSS, IL, USA).

### Ethics statement

Written informed consent was obtained from all patients. The study was conducted in accordance with the 1975 Declaration of Helsinki and approved by the Ethics Committee of the First Affiliated Hospital, School of Medicine, Zhejiang University, China.

## Results

### Basic characteristics of the research population

Of the total 503 HIV/AIDS patients, the following coinfections were reported: HIV/HBV (n = 55), HIV/HCV (n = 29), and HIV/HBV/HCV (n = 14). Patients were also affected by the following: COPD (n = 7), heart failure (n = 2), thyroid disorders (n = 3) and renal injury (n = 6). These patients were excluded; therefore, 387 patients were recruited in our cohort. Of these, 281 (72.6%) were male and 106 (27.4%) were female. The average age of the patients was 39.1±12.0 years. The average serum sodium concentration of the patients was 132.9±6.5 mmol/L. There were 206 patients (53.2%) with hyponatremia, including 5 (1.3%) with severe hyponatremia, 36 (9.3%) with moderate hyponatremia, and 165 (42.6%) with mild hyponatremia. Because of the low frequency of severe hyponatremia, these patients were placed into the moderate hyponatremia group for statistical analysis. The proportion of hyponatremia was 55.7% (59/106) among female patients, and 52.3% (147/281) among male patients (*P* = 0.965). The basic characteristics of the patients are shown in Table 1.

**Table 1:** Basic characteristics of the hyponatremic patients (n = 387).

Factor	Total n (%)	Sodium concentration Mean ± SD	*P* value	Median/Severe hyponatremia n (%)	Mild hyponatremia n (%)	Normonatremia n (%)	*P* value
**All**	387 (100)	132.9±6.5		41 (10.6)	165 (42.6)	181 (46.8)	
**Sex**							
Male	281 (72.6)	132.9±6.6	0.937	30 (7.8)	117 (30.2)	134 (34.6)	0.806
Female	106 (27.4)	132.9±6.3		11 (2.8)	48 (12.4)	47 (12.1)	
**Age [(39.1±12.0) years old]**							
<30	75 (19.4)	133.6±7.0	0.333	9 (2.3)	27 (7.0)	39 (10.1)	0.432
≥30	312 (80.6)	132.7±6.4		32 (8.3)	138 (35.7)	142(36.7)	
**WHO stage**							
I/II	61 (15.8)	136.7±5.4	<0.001	1 (0.3)	18 (4.7)	42 (10.9)	<0.001
III	138 (35.7)	132.8±6.6		18 (4.7)	53 (13.7)	67 (17.3)	
IV	188 (48.6)	131.7±6.3		22 (5.7)	94 (24.3)	72 (18.6)	
**CD4 count [47 (14**–**142) cells/µL]**							
Missing	50 (12.9)	131.9±5.3	<0.001	4 (1.0)	28 (7.2)	18 (4.7)	<0.001
≤50	172 (44.4)	131.0±7.1		30 (7.8)	83 (21.4)	59 (15.2)	
51–200	98 (25.3)	134.3±5.4		4 (1.0)	39 (10.1)	55 (14.2)	
≥201	67 (17.3)	136.5±5.3		3 (0.8)	15 (3.9)	49 (12.7)	
**HIV-RNA [(5.45±0.92) lg/mL]**							
Missing	189 (48.8)	132.4±5.9	0.352	21 (5.4)	90 (23.3)	78 (20.2)	0.128
<10^5^	56 (14.5)	133.5±8.5		8 (2.1)	17 (4.4)	31 (8.0)	
≥10^5^	142 (36.7)	133.3±6.5		12 (3.1)	58 (15.0)	72 (18.6)	
**Hemoglobin [(109.9±26.9) g/L]**							
Missing	4 (1.0)		<0.001	0	1 (0.3)	3 (0.8)	<0.001
<80	61 (15.8)	129.1±8.0		16 (4.1)	29 (7.5)	16 (4.1)	
80–109	122 (31.5)	132.4±5.7		12(3.1)	66 (17.1)	44 (11.4)	
≥109	200 (51.7)	134.4±6.0		13 (3.6)	69 (17.8)	118 (30.5)	
**Albumin [(29.2±7.8) g/L]**							
Missing	9 (2.3)		<0.001	0	3 (0.8)	6 (1.6)	<0.001
<30	187 (48.3)	130.4±6.6		34 (8.8)	99 (25.6)	54 (14.0)	
≥30	191 (49.4)	135.2±5.5		7 (1.8)	63 (16.3)	121 (31.3)	

### The serum sodium concentration was closely correlated with the progression of HIV/AIDS

The CD4 count and WHO stage are basic laboratory indicators of the progression of HIV/AIDS. The correlations among serum sodium concentration, CD4 count, and WHO stage were evaluated. In this study, the patients were stratified according to their CD4 count: ≤50 cells/µL, 51–200 cells/µL, and ≥201 cells/µL. The average serum sodium concentration was 131.0±7.1 mmol/L in patients whose CD4 count was 0–50 cells/µL, 134.3±5.4 mmol/L in patients whose CD4 count was 51–200 cells/µL, and 136.5±5.3 mmol/L in patients whose CD4 count was ≥201 cells/µL (F = 20.680, *P*<0.001). Pearson correlation analysis suggested that a decrease in serum sodium concentration was positively correlated with CD4 depletion (r = 0.292, *P*<0.001). The serum sodium levels and WHO clinical stage of HIV/AIDS were also evaluated. The serum sodium concentration was 136.7±5.4 mmol/L in patients in WHO stage I/II, 132.8±6.6 mmol/L in stage III and 131.7±6.3 mmol/L in stage IV (F = 14.586, *P*<0.001). The serum sodium concentration was negatively correlated with the WHO stage (r = −0.242, *P*<0.001).

**Figure 1:**
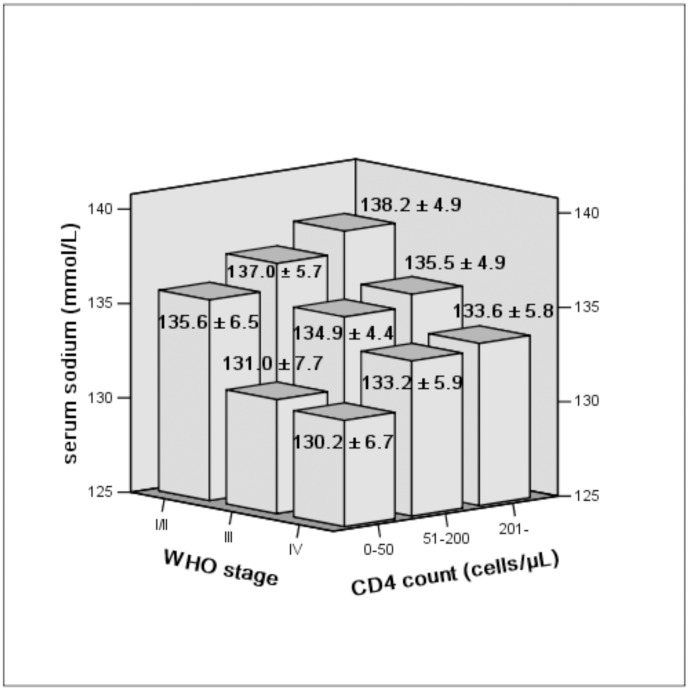
Mean serum sodium concentration in patients according to CD4 count and WHO stage. Serum sodium concentration decreases with a reduction in CD4 count and an increases with WHO stage (F = 7.004, *P*<0.001).

Furthermore, the progression of disease was evaluated together with the WHO stage and CD4 level, our research illustrated patients in advanced WHO stages and with a lower CD4 count had a lower serum sodium concentration, while the patients in the primary WHO stage and with a higher CD4 count had higher serum sodium concentration (F = 7.004, P<0.001) (Figure 1).

Additionally, HIV-RNA and the serum sodium concentrations were analyzed in our study. The serum sodium concentration in patients with HIV-RNA <10^5^ copies/mL was similar to that in patients with HIV-RNA >10^5^ copies/mL (133.5±8.5 *vs*. 133.3±6.5, *t* = 0.110, *P* = 0.913).

### Severe/moderate hyponatremia is an independent predictor of long-term survival of hospitalized AIDS patients

**Figure 2:**
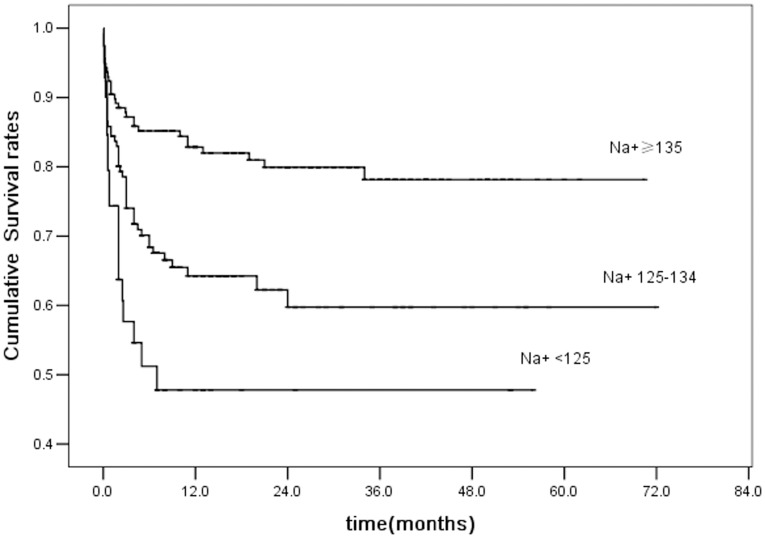
Kaplan–Meier survival curves according to serum sodium concentration. The serum sodium level affects mainly the 3-year mortality rate of patients. The 3-year cumulative survival rates for patients with moderate/severe and mild hyponatremia were 47.8%±8.5% and 59.8%±5.0%, respectively, and 78.2%±3.8% for normonatremic patients (Log-Rank, *P*<0.001).

The effect of serum sodium concentration on the long-term survival of patients was determined. During the follow-up time, 100 (25.8%) patients died, including 19 patients with moderate/severe hyponatremia, 49 with mild hyponatremia, and 32 with normonatremia. Of these, 86 patients (86.0%) died within 6 months of initial follow-up, eight (8.0%) died within 6–12 months, 3 (3.0%) died within 18–24 months, and three (3.0%) died within 24–36 months. Kaplan–Meier analysis indicated the 3-year cumulative survival rates were 47.8%±8.5% for patients with moderate/severe hyponatremia, 59.8%±5.0% for those with mild hyponatremia and 78.2%±3.8% for normonatremic patients (Log-Rank, *P*<0.001) (Figure 2).

Factors that may influence long-term survival were next analyzed. Patients were stratified according to the following criteria: age (years) (<30, ≥30), WHO stage (I/II, III, IV), CD4 count (cells/µL) (0–50, 51–200, ≥201), HIV-RNA (copies/mL) (<10^5^ copies/mL, ≥10^5^ copies/mL) and hemoglobin (g/L) (<80, 80–109, ≥110). Our data indicated that the following factors contribute to mortality: CD4 count, WHO stage, hemoglobin, albumin and serum sodium concentration. In the unadjusted model, the hazard ratios were 2.0 (95% CI: 1.3–3.1) times for patients with mild hyponatremia compared to those with normonatremia, and 3.4 (95% CI: 1.9–6.1) for patients with moderate/severe hyponatremia compared to those with a normal serum concentration. In multivariate Cox proportional hazard models adjusted for age, CD4 count, WHO stage, hemoglobin, and albumin, our data showed that compared to normonatremic patients, the relative hazard was 3.5 (95% CI: 1.9–6.5) for patients with moderate/severe hyponatremia (*P*<0.001) and 1.5 (95% CI: 0.9–2.4) for patients with mild hyponatremia (*P* = 0.161) (Table 2).

**Table 2:** Risk factors for mortality of patients in univariate/multivariate Cox's proportional hazard models.

Factors	Numbers (n = 387)	Deaths n (%)	Univarate model			Multivariate model		
			RH	95% CI	*P* value	RH	95% CI	*P* value
**Sex**					-			-
Male	281	67 (23.8)						
Female	106	33 (31.1)						
**Age(years)**								
<30	75	14 (18.7)	1.0			1.0		
≥30	312	86 (27.6)	1.5	0.8–2.6	0.193	2.5	1.1–5.5	0.025
**WHO stage**								
I/II	61	4 (6.6)	1.0			1.0		
III	138	24 (17.4)	2.8	1.0–8.1	0.056	2.0	0.6–6.8	0.271
IV	188	72 (38.3)	6.7	2.4–18.2	<0.001	3.7	1.1–12.2	0.031
**CD4 count**								-
Missing	50	16 (32.0)						
≤50	172	53 (30.8)	3.5	1.5–8.3	0.003			
51–200	98	25 (26.4)	2.9	1.2–7.1	0.019			
≥201	67	6 (8.9)	1.0					
**HIV-RNA**					-			-
Missing	189	46 (24.3)						
<10^5^	56	11 (19.6)						
≥10^5^	142	43 (30.3)						
**Hemoglobin**								
Missing	4	1						
<80	61	20 (32.8)	2.2	1.4–3.4	0.001	2.2	1.3–3.6	0.003
80–109	122	42 (34.4)	1.9	1.1–3.3	0.022	1.7	0.9–3.2	0.104
≥110	200	37 (18.5)	1.0			1.0		
**Albumin**								-
Missing	9	1						
<30	187	63 (33.7)	2.3	1.5–3.5	<0.001			
≥30	191	33 (17.3)	1.0					
**Serum sodium**								
** Normonatremia**	181	32 (17.7)	1.0			1.0		
** Mild hyponatremia**	165	49 (29.7)	2.0	1.3–3.1	0.003	1.5	0.9–2.4	0.161
** Severe/moderate hyponatremia**	41	19 (46.3)	3.4	1.9–6.1	<0.001	3.5	1.9–6.5	<0.001

## Discussion

Recent studies have suggested that serum sodium concentrations are a useful indicator of survival in liver cirrhosis patients [29], [30]. Hyponatremia is also common in AIDS patients; however, its precise effects in HIV/AIDS patients, especially their progression or prognosis, remains open to debate. Dao *et al*. reported research indicating high mortality in the first year of initiating HAART in a population of African female patients with both hyponatremia and hypochloremia [14]. However, a number of issues remain to be clarified, such as whether hyponatremia independently influences the long-term survival of HIV/AIDS patients, the effect of serum sodium concentration on patient mortality, the relationship between serum sodium concentration, CD4+ cell counts, the severity of HIV/AIDS and the impact of hyponatremia on patient survival in other populations. Thus, we conducted this study to determine the correlations of serum sodium concentration with the progression of HIV/AIDS and long-term survival.

Our data demonstrated that the overall frequency of hyponatremia was approximately 53.2% in HIV/AIDS patients; the serum sodium concentration of HIV/AIDS patients is strongly correlated with HIV/AIDS progression, and patients with severe/moderate hyponatremia (<125 mmol/L) were at higher risk of death during the long-term follow-up.

Hyponatremic patients are more susceptible to opportunistic illnesses or AIDS-related complex [5], [6]. Our study indicated that the overall incidence of hyponatremia in Chinese hospitalized patients was approximately 53%, which was obviously higher than the frequency of 38% reported by Tang et al. [5]. It can be speculated that the higher incidence of hyponatremia can be explained by a number of factors. First, a majority of subjects (84.2%) in our study were WHO stage III or IV (Table 1); therefore, most patients were complicated with severe opportunistic infections or AIDS-related complex. Second, serum sodium concentrations less than 135 mmol/L were defined as hyponatremic in our study, whereas Cusano *et al.* used a lower serum sodium concentration (<130 mmol/L) as the definition of hyponatremia [6].

The present study revealed a significantly positive correlation between serum sodium concentrations and the number of CD4+ cells and a negative correlation with the WHO clinical stage. These observations indicate that serum sodium concentrations can be used as an indicator of the progression of HIV/AIDS independent of the CD4 count and WHO clinical stage. Furthermore, although it is not a specific marker of AIDS, hyponatremia is recognized to be relevant to serious complications and increased mortality of AIDS patients [5], [6]. Thus, given that measurement of serum sodium concentrations is inexpensive and simple, this relatively objective marker could be used as a measure of AIDS severity in resource-limited countries or in remote rural areas of developing countries.

Our data also indicated that the serum sodium concentration affected the long-term survival of HIV/AIDS patients. Compared with normonatremic patients, hyponatremic HIV/AIDS patients were at a higher risk of death. In a universal model, patients with mild hyponatremia had a 2.0-fold higher risk of death compared with normonatremic patients, while the patients with severe/moderate hyponatremia were at 3.4-fold higher risk of death than normonatremic patients. Previous research has indicated that albumin [31], hemoglobin [32], CD4 count [33]and CD4 percentage [34] are closely linked with the mortality of AIDS patients. Our study further showed that after adjustment for age, WHO stage, CD4 count, hemoglobin and albumin in a multivariate model, severe/moderate hyponatremia remained a high risk factor for death. These data are consistent with the meta-analysis of results in commonly observed clinical conditions across large numbers of patients [35] and furthermore, illustrated that the serum sodium concentration reflects the severity and mortality of Chinese hospitalized HIV/AIDS patients. This suggests that earlier and intensive medical management is necessary to improve the outcome of such patients.

The survival of patients during the follow-up period was less affected by the serum sodium concentration after the initial 3 years (Figure 2); antiretroviral therapy (ART) and other treatments were likely to be responsible for this phenomenon. The introduction of ART drugs resulted in a significant reduction in AIDS-related mortality [36]–[38]. In our research, most AIDS patients (84.2%) were in stage III or IV (Table 1), and accepted ART when their CD4+ cells counts were <200 cells/µL. Thus, the administration of ART and elimination of opportunistic infections were likely the major reasons for survival beyond 3 years.

A number of complex factors predispose a patient to hyponatremia, including fluid loss due to diarrhea or vomiting [5], adrenocortical insufficiency [12], pulmonary and CNS infection that may be accompanied by SIADH [9], [11]. Previous studies have suggested that some CNS infections, such as cryptococcal meningitis [39] or tuberculosis meningitis [40], [41] result in a low serum sodium concentration. Pneumonia is also a common reason for the development of hyponatremia [11]. In our study, most patients (84.2%) were WHO stage III or IV; therefore, most of our patients were suffering severe opportunistic infections, such as pulmonary and CNS infections, or malignant diseases. Although the serum level of the antidiuretic hormone was not measured, the clinical characteristics of most patients were consistent with SIADH caused by intracranial or pulmonary infections. We, therefore, propose that SIADH is one of the major causes of hyponatremia. In addition, diarrhea associated with gastroenteric complications may have an impact on hyponatremia due to fluid loss.

Our study did not demonstrate a direct association between HIV-RNA and hyponatremia. The probable reasons for this were investigated. First, patient autopsies or biopsy indicated that there were no significant differences in the number of vasopressin-expressing neurons in the paraventricular nucleus of the hypothalamus between HIV-infected patients and the controls [42], and HIV infection of the pituitary is uncommon [4]. These observations indicated that HIV does not directly affect the hypothalamus and pituitary. Second, in vitro studies suggest that HIV interaction with arginine-vasopressin producing neurons are dependent on gp120 and independent of HIV-RNA [43]. Third, HIV-RNA assays were not performed in almost 50% of the patients in our study, thus, the relationship between HIV-RNA and hyponatremia were not precisely evaluated. Thus, further research is required to clarify the association between HIV-RNA and hyponatremia.

In summary, our data demonstrate an increased frequency of hyponatremia (approximately 53.2%) in Chinese hospitalized HIV/AIDS patients, and that serum sodium concentrations are associated with the CD4+ cell count and clinical stage of HIV/AIDS patients. Hyponatremia is associated with poor outcomes in hospitalized HIV/AIDS patients, but only moderate/severe hypontremia appears to increase the risk of long-term mortality. Thus, serum sodium is implicated as a useful indicator of long-term survival in Chinese hospitalized AIDS patients.

However, some deficiencies in present study should be noted. First, our study was not conducted in a large populations. Second, we were unable to measure the antidiuretic hormone of patients to further confirm our results. Third, we were unable to evaluate the specific effects of HIV-RNA on patient survival because of the lack of HIV-RNA data in a large proportion of the participants. Therefore, more detailed prospective studies in larger populations are required to determine to what extent HAART can correct this disorder, and the effect of sodium supplementation on the long-term outcomes of HIV/AIDS patients with hyponatremia.
